# Accurate Estimation of the Standard Binding Free Energy of Netropsin with DNA

**DOI:** 10.3390/molecules23020228

**Published:** 2018-01-25

**Authors:** Hong Zhang, Hugo Gattuso, Elise Dumont, Wensheng Cai, Antonio Monari, Christophe Chipot, François Dehez

**Affiliations:** 1 Research Center for Analytical Sciences, College of Chemistry, Tianjin Key Laboratory of Biosensing and Molecular Recognition, Nankai University, Tianjin 300071, China; hzhang@mail.nankai.edu.cn (H.Z.); wscai@nankai.edu.cn (W.C.); 2 UMR 7019, Theoretical Physics and Chemistry Department (LPCT), Université de Lorraine-Nancy, 54506 Vandoeuvre-lès-Nancy, France; hugo.gattuso@univ-lorraine.fr (H.G.); Antonio.Monari@univ-lorraine.fr (A.M.); Christophe.Chipot@univ-lorraine.fr (C.C.); 3 UMR 7019, Theoretical Physics and Chemistry Department (LPCT), CNRS, 54506 Vandeouvre-lès-Nancy, France; 4 Univ Lyon, Ens de Lyon, CNRS UMR 5182, Laboratoire de Chimie, Université Claude Bernard Lyon 1, F-69342 Lyon, France; elise.dumont@ens-lyon.fr; 5 Collaborative Innovation Center of Chemical Science and Engineering, Nankai University, Tianjin 300071, China; 6 Laboratoire International Associé Centre National de la Recherche Scientifique et University of Illinois at Urbana-Champaign, Champaign, Illinois, 54506 Vandeouvre-lès-Nancy, France; 7 Department of Physics, University of Illinois at Urbana-Champaign, 1110 West Green Street, Urbana, IL 61801, USA

**Keywords:** binding free energy, DNA sensitization, netropsin, all-atom molecular dynamics, minor-groove binder

## Abstract

DNA is the target of chemical compounds (drugs, pollutants, photosensitizers, etc.), which bind through non-covalent interactions. Depending on their structure and their chemical properties, DNA binders can associate to the minor or to the major groove of double-stranded DNA. They can also intercalate between two adjacent base pairs, or even replace one or two base pairs within the DNA double helix. The subsequent biological effects are strongly dependent on the architecture of the binding motif. Discriminating between the different binding patterns is of paramount importance to predict and rationalize the effect of a given compound on DNA. The structural characterization of DNA complexes remains, however, cumbersome at the experimental level. In this contribution, we employed all-atom molecular dynamics simulations to determine the standard binding free energy of DNA with netropsin, a well-characterized antiviral and antimicrobial drug, which associates to the minor groove of double-stranded DNA. To overcome the sampling limitations of classical molecular dynamics simulations, which cannot capture the large change in configurational entropy that accompanies binding, we resort to a series of potentials of mean force calculations involving a set of geometrical restraints acting on collective variables.

## 1. Introduction

DNA is constantly exposed to various sources of stress, which may ultimately damage its chemical composition [1,2,3,4,5,6,7,8], a situation particularly deleterious for biological cells [9,10]. If not properly repaired [11] DNA damages can induce either the cellular death, via necrosis or apoptosis, or, lead to mutations [12,13] that in superior organisms may lead to cancerogenesis [1,14]. DNA lesions can result both from endogenous and exogenous sources and comprise oxidative stress and exposure to UV light [1,4,15,16,17,18]. Among oxidative stress inducers one can cite the reactive oxygen species such as singlet oxygen (${}^{1}$O${}_{2}$) [19,20] or hydroxyl (HO·) and peroxide (HOO·) radicals [21]. The absorption or UVB and UVA light by the strongly coupled DNA nucleobases triggers instead complex photochemical pathways, which, despite the inherent DNA photostability [22,23,24,25], may result in the accumulation of pyrimidine and especially thymines dimers. Among the most common and dangerous lesions are the cyclobutane pyrimidine dimers (CPD) and the 6-4 photoproduct (64-PP). The already intricate scenario becomes even more complex when one takes into account the interaction between DNA and external drugs. In this case, a first distinction should be made among covalent and non-covalent DNA binders [7,26,27]. Non-covalent binders form supramolecular aggregates, with the interaction being driven by electrostatic and dispersive (π-stacking) interactions. Non-covalent DNA adducts can be highly stable and persistent, and, in some cases, lead to replication blockage and apoptosis. In addition to this role, they can act as photosensitizers and exacerbate the toxicity of other stress sources, such as UV light or ionizing radiation [26,28,29,30,31,32,33,34,35]. Sensitizers can act either via the production of ${}^{1}$O${}_{2}$ [36], via energy transfer, usually from the triplet manifold [28,30,37,38], or via electron transfer, usually towards guanine [39,40]. Moreover, photosensitizers may also trigger photochemical reactions, leaving reactive radicals in close proximity to the DNA, capable of further inducing deleterious reactions such as hydrogen abstraction or strand breaks [29,35,41].

The photosensitization mechanism and its outcome strongly depend on the interaction mode of the DNA macromolecule and the drug. However, photosensitizers may exhibit multiple binding motifs in competition between each other [37,42]. A non-covalent DNA binder may interact either via the major or the minor groove, or slip in between base pairs to give rise to intercalation. In other cases, ejection of one of the Watson-Crick paired nucleobases from the DNA helical structure may occur (insertion). The ejection of a full base pair (double-insertion) has also been reported for organic and organometallic interactors [37]. Furthermore, a given drug may give rise to multiple interaction modes, the equilibrium of which can depend upon the DNA sequence, as well as on environmental factors (salt concentration, crowding, etc.). A stunning example is the paradigmatic sensitizer benzophenone for which the structure of its DNA aggregate has been obtained only thanks to molecular modeling [37]. Obviously, the absence of precise structures complicates enormously the study and rationalization of DNA/drug interactions and of their (photo-) reactivity. In that respect, molecular modeling and simulations provide an unprecedented atomistic resolution, allowing to precisely tackle the persistence of different interaction modes, as well as the fine coupling between the different potentially reactive moieties. This is particularly true thanks also to the development of always more accurate force fields able to reproduce nucleic acids dynamics. Furthermore, when coupled with quantum chemistry, quantum mechanics/molecular mechanics (QM/MM) hybrid simulations may provide a complete rationalization of the subsequent (photochemical) reactivity, as well as of the role played by the molecular environment in tuning them [43,44]. However extremely powerful, conventional equilibrium molecular dynamics (MD) does not allow to discriminate between the different stability of competitive binding modes. In particular, equilibrium sampling is not able to capture the large change in configurational entropy related to binding and may lead to excited conformations trapped in a higher minimum region of the free energy landscape. To palliate this disadvantage, biasing techniques should be used in order to ensure proper sampling of the conformational space and, thus, obtain accurate binding free energies for different aggregates and different binding modes. The last years have seen a surge of results from binding free-energy calculations of ligand-protein or even protein-protein complexes. This is due both to the steep increase of the computational capacity of supercomputers and to the development of specifically tailored and efficient sampling techniques. However, the situation is much less well defined in the case of DNA interactions. Most of the studies have hitherto dealt with approximate methods, usually relying on an implicit description of the molecular environment. The popular MM/GBSA approach belongs to this class of methods and has been used to obtain a rough estimate of the binding free energy for a variety of complexes [44,45,46,47,48]. The computed quantities inherently depend on stringent choices and empirical parameters such as the dielectric constant, the Van der Waals radii and the approximation of the entropic contribution. For this reason, electrostatics-based approximate methods are often poorly predictive, but may prove useful for fast discrimination of poses in high-throughput docking [49]. Alternatively, more rigorous, statistical mechanics-based methods can been applied [50], including thermodynamic integration [51] and free-energy perturbation [52]. Accurate estimation of standard DNA-ligand binding free energies remains a daunting computational challenge. Recently, we have developed an original framework based on first principles eliciting protein-ligand and protein-protein absolute binding free energies to be determined with utmost reliability [53,54]. The proposed strategy has been enhanced with the introduction of tailored coarse variables, germane to virtually any host-guest complexes [55]. Here, we probe this methodology to the challenging case of DNA-ligand recognition and association.

In this contribution, we turn to a very well-known and highly specific minor groove binder, namely netropsin [46,56,57], the binding free energy with DNA of which has been precisely determined by Breslauer and coworkers [58] in the eighties. Netropsin being a minor-groove binder, it has a particular affinity for AT-rich DNA regions, and calorimetric and spectroscopic titrations have shown that it prefers to bind with an alternating poly(dAdT)-poly(dAdT) polymer (−12.7 kcal/mol) than a poly(dA)-poly(dT) homopolymer (−12.1 kcal/mol), while the binding free energy for a mixed GCGAATTCGC sequence is of −11.5 kcal/mol. Kopka et al. attributed the preference toward poly-AT double-stand DNA not to hydrogen bonding but rather to close van der Waals contacts between adenine C-2 hydrogens and CH groups on the pyrrole rings of netropsin [59]. Here, as a proof of concept, we demonstrate the possibility to determine with optimal accuracy the binding free energy of netropsin with an alternating poly(dAdT)-poly(dAdT) double strand, employing a series of potentials of mean force (PMF) and a series of geometrical coordinates to bias sampling. Special attention is devoted to the presence of different netropsin minor-groove binding conformations involving differences in the amino-group orientation and, hence, in the emergence of the netropsin/DNA specific interaction networks.

## 2. Results and Discussion

### 2.1. Dynamics of the Netropsin/DNA Complex

**Figure 1:**
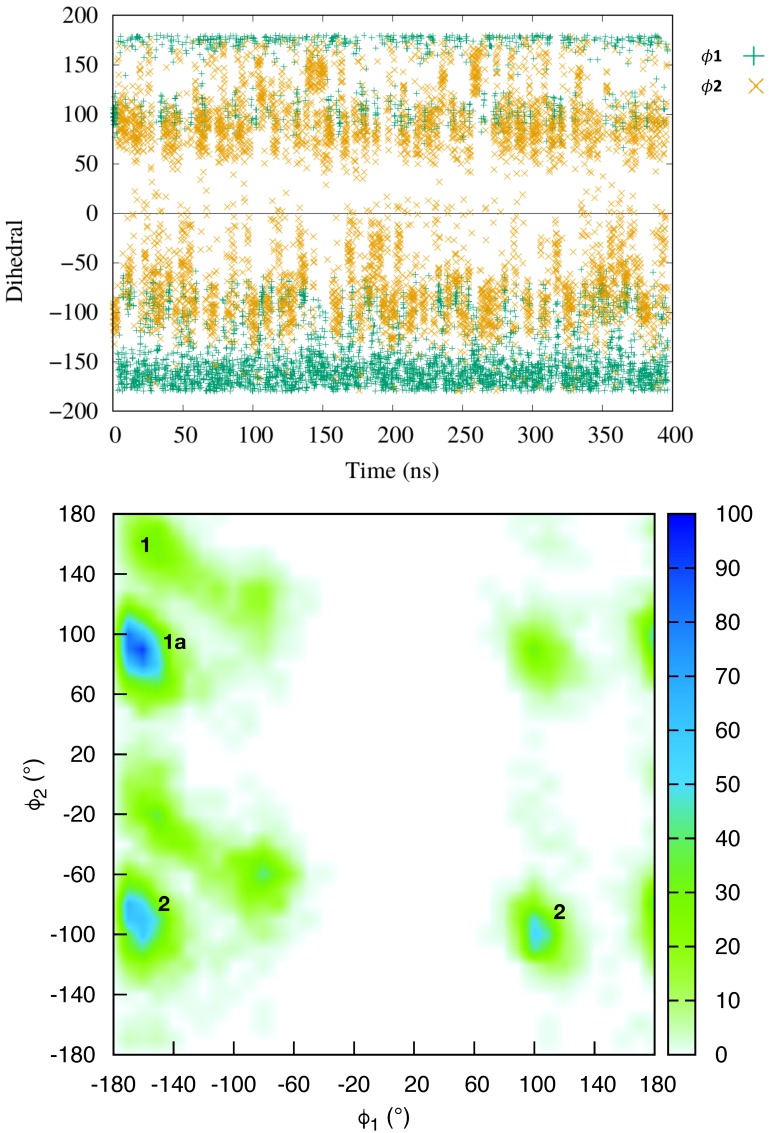
(**Top**) Time series of the representative dihedral angles $\phi_{1}$ (in green) and $\phi_{2}$ (in yellow) over the equilibrium MD trajectories; (**Bottom**) Distribution of the $\phi_{1}$ and $\phi_{2}$ dihedral angles over the trajectory.

The equilibrium molecular dynamics have resulted in a persistent complex between netropsin and DNA, whereby the ligand remained bound to the minor groove, consistently with previous studies [46,60]. The conformational dynamics of the DNA within the complex follows that of an isolated B-DNA double strand. The netropsin is globally rigid, but a closer inspection reveals that the ligand is in a conformational equilibrium between structures exhibiting different orientations of the ligand terminal cationic group, involved in the DNA binding. To characterize this conformational exchange we have followed the time evolution of two dihedral angles ($\phi_{1}$ and $\phi_{2}$) of the netropsin scaffold (see Figure 1).

**Figure 2:**
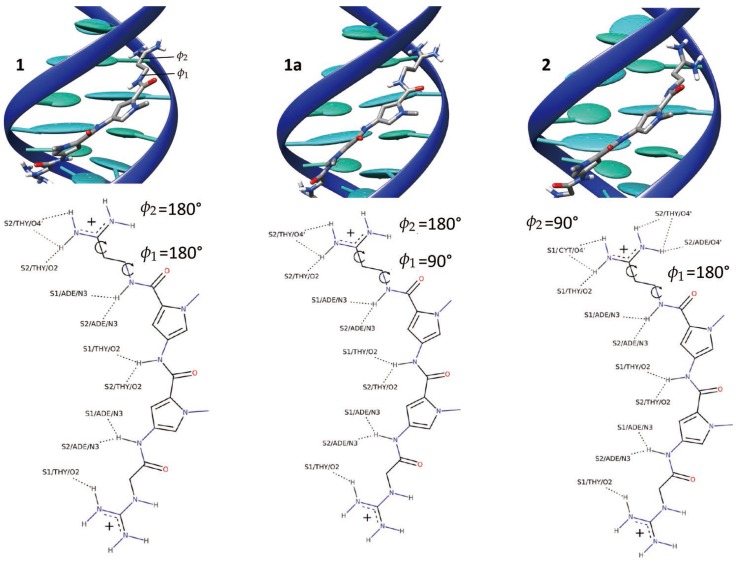
Representative snapshots illustrating the 1, 1a and 2 binding mode of netropsin with B-DNA. The main interactions developing in the three modes, as well as the characteristic values of the $\phi_{1}$ and $\phi_{2}$ dihedral are also reported.

The time series of $\phi_{1}$ and $\phi_{2}$ (Top panel of Figure 1) shows that in the course of the simulation, the netropsin is exchanging constantly and rapidly between different conformers. The 2D plot of the population of ($\phi_{1}$,$\phi_{2}$) conformers (Bottom panel of Figure 1) reveals that the netropsin can adopt three well-defined conformations, e.g., 1, 1a and 2, which are represented in Figure 2 together with their interaction network with DNA. Conformer 1 corresponds to a flat geometry ($\phi_{1}∼\phi_{2}∼\pm 180^{\circ }$). The conformation 1a only differs from 1 by the out of plane organization of the -CH${}_{2}$-CH${}_{2}$- aliphatic chain ($\phi_{1}∼\phi_{2}∼\pm 90^{\circ }$). As far as the interaction with DNA is concerned, conformers 1 and 1a forms the same binding pattern. In conformer 2, the guanidinium moiety of netrospin is oriented perpendicularly to the rest of the ligand ($\phi_{1}∼\pm 180^{\circ },\phi_{2}∼\pm 90^{\circ }$) and can no longer interact with the minor groove.

From the analysis of 5000 snapshots taken regularly along the entire equilibrium simulation, conformation 1, 1a and 2 accounts for 6.36%, 30.44% and 45.40% of the total population, respectively (Assuming a threshold of $45^{\circ }$ around the most populated region). In addition 17.8% of the structures are transition conformations, a consequence of the fast and constant switching between the three conformers. Globally, our data suggest a relatively equilibrated population of both 1 + 1a and 2 conformations, in line with the experiments carried out by Lewis et al. [56] who reached the conclusion that netropsin populates equivalently two binding configurations at a single binding site. Furthermore, over the 11 NMR structures obtained by Rettig et al. [57], conformer 1 is found 3 times, 1a 3 times and 2 5 times which correlates again nicely with our theoretical observations.

### 2.2. Absolute Binding Free-Energy of Netropsin to DNA

We calculated the standard binding free energy using geometrical transformations with the new coarse variables [55]. In the present work, we turned to the extended adaptive biasing force (eABF) algorithm [61,62] to compute the different free-energy profiles of the thermodynamic cycle that underlies binding. The particular example of netropsin bound to B–DNA is particularly well-suited for this geometric route to standard binding free energies owing to the binding mode of the guest, which remains at the surface of the nucleic acid. This binding pose is at variance with intercalated motifs, wherein the guest molecule is interred in the double strand, thereby precluding the use of a separation PMF with restrained orientational, conformational and positional degrees of freedom. In the latter case, an alternate alchemical route [53,63], whereby the guest is decoupled reversibly from its environment, namely the host and the aqueous medium, ought to be preferred. This strategy has been cogently illustrated in the instance of benzophenone bound to a DNA double strand [42].

**Figure 3:**
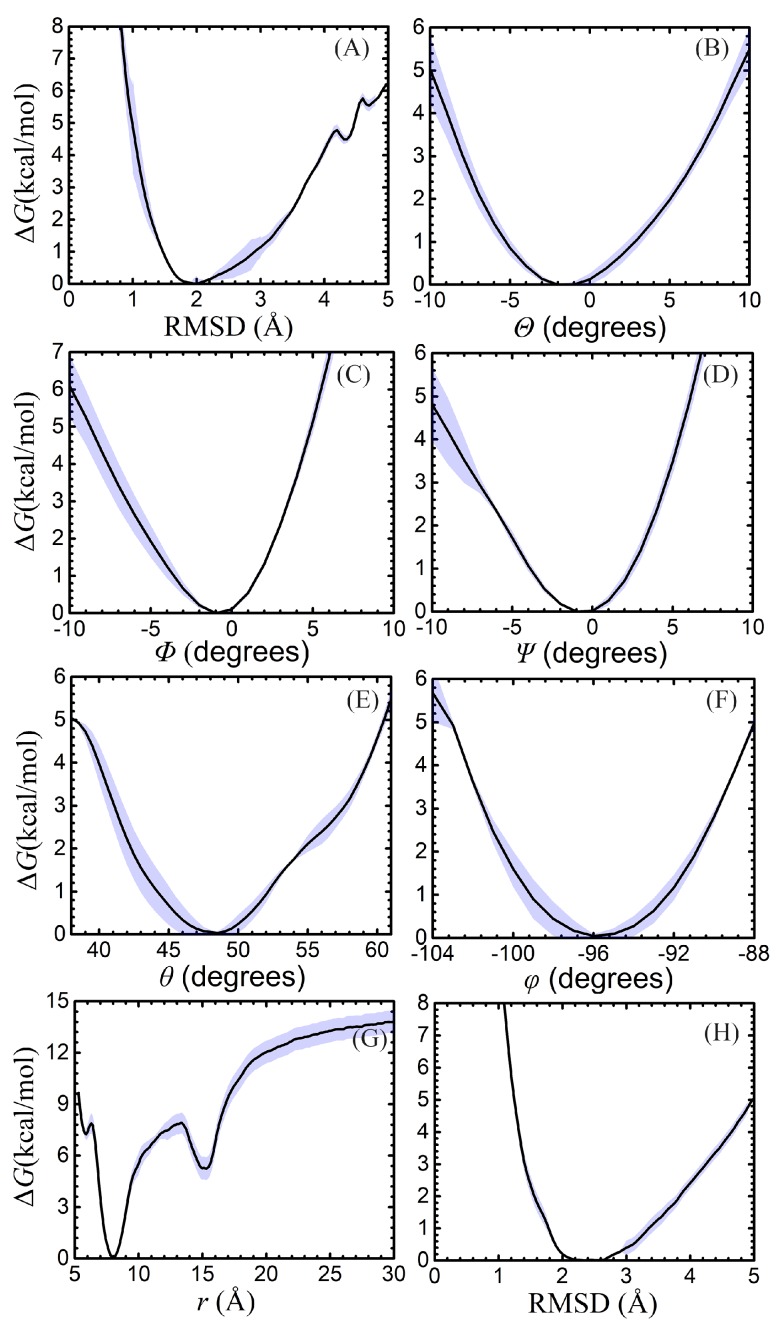
PMFs corresponding to each contribution of Table 1. Plots for the RMSD in the bound state and in the bulk in **A** and **H**, for the three Euler angles, Θ, Φ, and Ψ are given in **B**–**D**, respectively, for the positional restraints on θ and φ in **E** and **F**, for the separation in **G**. The error bars are showed in grey.

The results of the free-energy calculations are gathered in Table 1, and the one-dimensional free-energy profiles for the different contributions are reported in Figure 3. The theoretical estimate of the standard binding free energy of netropsin and B-DNA is −13.2 kcal/mol, which is in good agreement with the experimental value of −12.7 kcal/mol, reported by Breslauer and coworkers for an alternate poly(dAdT)–poly(dAdT) seuence [58]. The statistical error for the different free-energy contributions has been simply estimated by dividing the statistical data of the simulations into two blocks of equal size. The error bars for the individual PMFs amount to about ±1.6, ±1.2, ±0.0, ±0.0, ±0.0, ±0.0, ±0.0 and ±0.6 kcal/mol for both unbound- and bound-state RMSD terms, the five angular terms, Θ, Φ, Ψ, θ, φ, and the separation term, respectively. Considering the flexibility of the double-stranded B-DNA segment, a relatively larger force constant has been used to restrain the conformation of DNA, comparatively with the other terms. We have shown that the standard binding free energy is independent of the choice of the force constant, provided that the latter is used consistently across the thermodynamic cycle underlying host–guest association [53]. The larger force constant and the marked flexibility of the B–DNA double strand rationalize the observed amplitude of the error associate to the RMSD terms.

**Figure 4:**
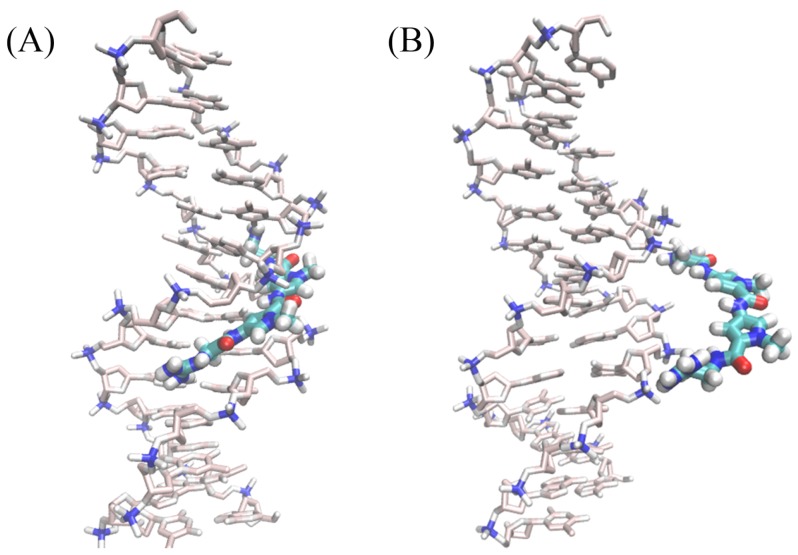
Snapshots extracted from the separation simulations for the double-well shape. (**A**) the representative structure near the first minimum; (**B**) the representative structure near the second minimum.

A rapid inspection of the free-energy contributions of Table 1 combined with Figure 3 confirms that the angular contributions are nearly negligible and that the corresponding PMFs are quadratic [53,54].As shown in Table 1, the free-energy calculations of angular contributions converge within 70 ns. This fast convergence rate can be ascribed to the limited change in position and orientation of the ligand in the bound state. Convergence of the PMF calculations for the conformation of the DNA in the bound and unbound states is more difficult to achieve on account of the large conformational space available to the double-stranded B-DNA. Interestingly enough, the harmonic nature of the free energy is also mirrored in the RMSD contributions. Conversely, the separation PMF has a noteworthy double-well shape, wherein the first minimum correpsonds to the native binding of netropsin to B–DNA (see Figure 4A) , whereas the second, shallower minimum reflects the possibility of the charged guest to form non-native contacts with host along the rectilinear separation path (Figure 4B). It is worth mentioning that the latter is purely arbitrary and does not correspond to a minimum-action path. However, the observed second minimum is indicative of an complex driven only by electrostatic interactions with the negatively charged DNA backbone, while netropsin is not yet inserted into the minor groove. Consistent with previous work [55], separation of the ligand from the DNA is the most computationally intensive step. For the reference non-interacting complex, we chose a distance of 30 Å from the DNA barycenter. Extending this distance only slightly affects the calculated free-energy difference [55].

## 3. Conclusions

In this article, we have determined with unprecedented accuracy the standard binding free energy of a small drug associated to the minor groove of a B–DNA double strand in explicit solvent. Towards this end, use was made of a computational strategy put forth recently and applied to protein–ligand [53] and protein–protein binding [54]. Owing to the considerable change in configurational entropy that accompanies host–guest association, which equilibrium brute-force molecular dynamics is not able to capture, the proposed strategy rests on the introduction of geometric restraints and the accurate evaluation of the loss of entropy arising from these restraints by means of PMF calculations along tailored collective variables [55].

While relative binding free-energy calculations have been popular for several years, owing to an advertised lesser computational investment, determination of standard binding free energies has remained hitherto scarce. It is noteworthy that under certain circumstances, notably in the concomitant mutation of several residues, estimation of the differential binding free energy between two hosts through independent absolute binding free-energy calculations has proven to converge faster than a relative binding free-energy calculation [55]. Although the strategy utilized herein dramatically reduces the difficulty to sample the change in configurational entropy associated to binding, accurate evaluation of certain contributions to the free energy remain challenging, chief among which is the conformational term determined by means of a distance RMSD with respect to the native state. This challenge is mirrored in the staggering error bars reported in Table 1, and is rooted in the plasticity of B–DNA in an aqueous environment. It is worth mentioning that in contrast with the protein–ligand problem, wherein the host, i.e., the protein, is generally unrestrained and the conformation of the guest, i.e., the ligand, is coerced to that in the native state, in the present example of netropsin binding a B–DNA duplex, the latter, i.e., the host, is restrained to an equilibrated crystallographic structure, while the guest, i.e., netropsin, is free to isomerize. In practice, binders of the minor groove are sufficiently rigid to justify the absence of specific geometric restraints for the guest. In the particular case of netropsin, exchange between the different conformations is fast enough to be adequately sampled at thermodynamic equilibrium. The present work offers an extension to DNA association of the theoretical framework developed recently for the precise estimation of binding free energies involving protein hosts. It also paves the way for the estimation of standard binding free energies of complexes involving proteins and nucleic acids.

## 4. Theoretical Background

Accurate prediction of the standard binding free energy that underlies host–guest association represents a formidable computational challenge, and, under many circumstances, an Augean task. The difficulty of this endeavor can be understood in terms of the considerable change in configurational entropy that accompanies binding of the guest to the host, which equilibrium simulations cannot readily capture. Sampling of the relevant movements of the guest with respect to the host requires an elaborate workflow [53,63,64,65,66,67], wherein introduction of suitable geometrical restraints alleviates the intrinsic limitations of unbiased molecular dynamics. Under these premises, we have devised two distinct approaches for the determination of the standard binding free energy of host–guest association, invoking either alchemical, or geometric transformations, as a function of the problem at hand [53,54]. Each route has admittedly its own advantages and drawbacks, the former, which relies on the uncoupling of the guest from the host, being better suited for substrates interred in the binding pocket than the latter. On the other hand, owing to sampling limitations, the alchemical route is restricted to small guests.

**Figure 5:**
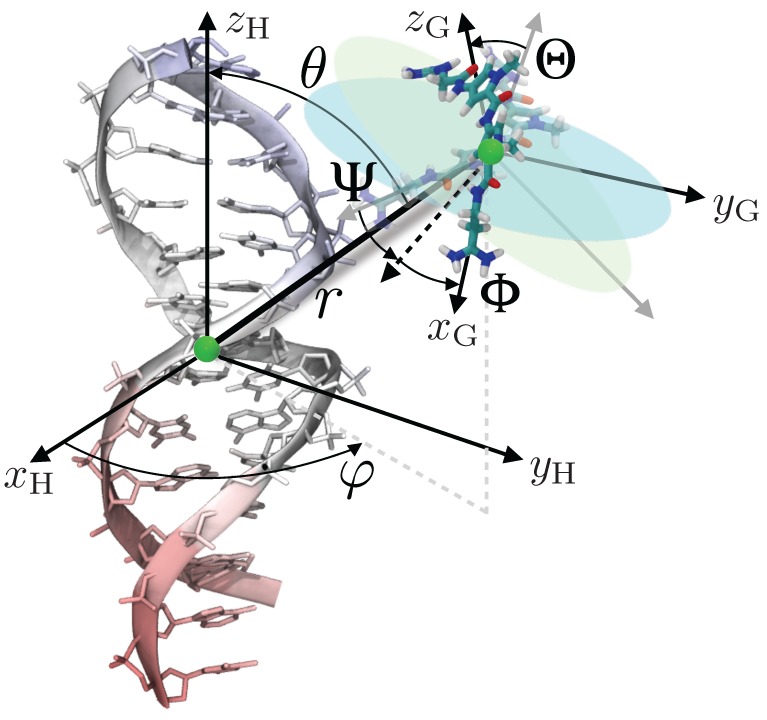
Degrees of freedom considered in the binding free-energy calculation illustrated in the case of netropsin binding to a double-stranded B-DNA segment. The Euler angles, Θ, Φ and Ψ, and the spherical-coordinate angles, θ and φ, describe the relative orientation and position of the guest with respect to the host, respectively.

Here, use will be made of the geometric route to determine the standard binding free energy of netropsin–DNA association, turning to a series of potential-of-mean-force (PMF) calculations. This approach presupposes a simplified representation of the inherently multidimensional reaction coordinate that describes host–guest association, and the introduction of geometrical restraints acting on the spatial degrees of freedom available to the guest. To enhance sampling further and, hence, improve convergence of the free-energy calculations, conformation of the guest is traditionally restrained. In the present work, the relative rigidity of netropsin obviates this requirement. Conversely, the marked flexibility of the double-stranded B-DNA segment imposes that its conformation be restrained to circumvent sampling inefficiency. The loss of configurational entropy due to the geometrical restraints, which necessarily impacts the standard binding free-energy, is accounted for rigorously in independent PMF calculations carried out for each restrained degree of freedom. In practice, the relative position and orientation of the guest are defined in the frame of reference of the host by means of, respectively, the two polar angles, θ and φ, and the three Euler angles, Θ, Φ and Ψ (see Figure 5). These angular degrees of freedom are introduced in the molecular dynamics simulations in the form of coarse variables, upon which a harmonic potential acts. Until recently, description of the position and orientation of the guest with respect to the host required the explicit definition of groups of atoms in both the latter and the former. Development of new coarse variables [55] describing the relative orientation and position of the guest through a global macromolecular orientational procedure obviates this requirement.

The three-dimensional structure of the B-DNA double strand was restrained to an average conformation through its distance root mean square deviation (RMSD) with respect to the latter. Geometrical restraints were then imposed sequentially on the five angular degrees of freedom based on the equilibrium geometry of the complex formed by netropsin bound to DNA. The free-energy cost incurred in the application of these geometrical restraints was determined in a stepwise fashion in six different PMF calculations. Last, the guest was separated reversibly from the host, following an ad-hoc rectilinear pathway, in a final one-dimensional free-energy calculation, wherein all other degrees of freedom are frozen to their equilibrium value in the bound state. Put together, seven independent PMF calculations were carried out on the netropsin–DNA complex in its bound state. For consistency, geometrical restraints imposed in the bound state ought to be also accounted for in the unbound state. Since the free-energy cost involved in the reorientation and translation of the quasi-rigid guest can be evaluated analytically, only one additional PMF calculation remains to be performed, namely that of the host in its free state. The equilibrium constant underlying netropsin–DNA association is then computed as a product of ratios of configurational integrals:

(1) $$\begin{array}{ccc} K_{\mathrm{eq}} & = & \frac{\int_{\mathrm{site}}^{\;}d1\int dx\;e^{-\beta U}}{\int_{\mathrm{bulk}}^{\;}d1\;\delta \left(x_{1}-x_{1}^{*}\right)\;\int dx\;e^{-\beta U}}\\ & = & \frac{\int_{\mathrm{site}}^{\;}d1\int dx\;e^{-\beta U}}{\int_{\mathrm{site}}^{\;}d1\int dx\;e^{-\beta (U+u_{c})}}\\ & \times & \frac{\int_{\mathrm{site}}^{\;}d1\int dx\;e^{-\beta (U+u_{c})}}{\int_{\mathrm{site}}^{\;}d1\int dx\;e^{-\beta (U+u_{c}+u_{o})}}\\ & \times & \frac{\int_{\mathrm{site}}^{\;}d1\int dx\;e^{-\beta (U+u_{c}+u_{o})}}{\int_{\mathrm{site}}^{\;}d1\int dx\;e^{-\beta (U+u_{c}+u_{o}+u_{a})}}\\ & \times & \frac{\int_{\mathrm{site}}^{\;}d1\int dx\;e^{-\beta (U+u_{c}+u_{o}+u_{a})}}{\int_{\mathrm{bulk}}^{\;}d1\;\delta \left(x_{1}-x_{1}^{*}\right)\;\int dx\;e^{-\beta (U+u_{c}+u_{o})}}\\ & \times & \frac{\int_{\mathrm{bulk}}^{\;}d1\;\delta \left(x_{1}-x_{1}^{*}\right)\;\int dx\;e^{-\beta (U+u_{c}+u_{o})}}{\int_{\mathrm{bulk}}^{\;}d1\;\delta \left(x_{1}-x_{1}^{*}\right)\;\int dx\;e^{-\beta (U+u_{c})}}\\ & \times & \frac{\int_{\mathrm{bulk}}^{\;}d1\;\delta \left(x_{1}-x_{1}^{*}\right)\;\int dx\;e^{-\beta (U+u_{c})}}{\int_{\mathrm{bulk}}^{\;}d1\;\delta \left(x_{1}-x_{1}^{*}\right)\;\int dx\;e^{-\beta U}}\\ & = & e^{-\beta \left(\Delta G_{c}^{\mathrm{site}}+\Delta G_{o}^{\mathrm{site}}+\Delta G_{a}^{\mathrm{site}}-\frac{1}{\beta }\ln\left(S^{*}I^{*}C^{\circ }\right)+\Delta G_{o}^{\mathrm{bulk}}+\Delta G_{c}^{\mathrm{bulk}}\right)} \end{array}$$

where 1 denotes the guest, netropsin, $x_{1}$, the position of its center of mass, and $x_{1}^{*}$, an arbitrary location in solution, sufficiently far from the binding site. *U* is the potential energy, $u_{o}=u_{\Theta }+u_{\Phi }+u_{\Psi }$ is the orientational restraining potential, and $u_{a}=u_{\theta }+u_{\varphi }$, the positional restraining potential. The standard binding free energy host–guest association is then computed as:

(2) $$\Delta G_{\mathrm{bind}}^{\circ }=-\frac{1}{\beta }\times \ln\;K_{\mathrm{eq}}C^{\circ }$$

where $C^{\circ }=\frac{1}{1661}$ Å${}^{3}$ is the standard, 1M concentration.

All the free-energy calculations in this contribution were performed using the extended adaptive biasing force (eABF) algorithm [61,62].

## 5. Materials and Methods

Force Field Parameterization Netropsin was optimized at density functional theory (DFT) B3LYP/ 6-311+G(d,p) level in a dielectric continuum (PCM) to reproduce water solvation, using Gaussian09 software [68]. Netropsin force field’s bound terms have been represented using generalized amber force field (GAFF) following a satisfactorily procedure for similar organic compounds. Atomic charges were subsequently obtained using the [69] procedure at the HF/6-31G* level of theory REF3. The bounded and non-bounded parameters were the amber99 force field including bsc1 correction [70] for DNA, and TIP3P parameters for water molecules [71].

Equilibrium Molecular Dynamics The starting configuration of the 14 base-pair-long poly(dA-dT)– poly(dA-dT) double-stranded B-DNA was created using the NAB utility of Ambertools16 [72]. Its complex with netropsin was created manually by placing the quantum-chemically optimized structure of the ligand in a comparable position and orientation as in the NMR experimental results by Rettig et al. [57] (pdbdatabank code: 2LWH). B-DNA/netropsin and B-DNA systems were placed in cubic boxes of respectively 70 Å (10,712 water molecules and 24 Na${}^{+}$ counterions) and 75 Å edge dimensions (12,983 water molecules and 26 Na${}^{+}$ counterions). The setups were prepared using the Antechamber and Leap Ambertools16 utilities, and the simulations were run using the NAMD molecular dynamics code version 2.12 [73]. The system was first relaxed using 1000 steps of energy minimization, using the conjugate-gradient algorithm, followed by three restrained molecular dynamics runs of 600 ps each, applying on heavy atoms respectively 100%, 50% and 10% of the geometric restraints, while allowing the relaxation of the solvent. A production run of 400 ns was then performed. Each MD simulation was run with a 2 fs time step under periodic boundary conditions. Temperature and pressure were held constant using Langevin dynamics and the Langevin piston [74,75]. Nonbonded van der Waals interactions were truncated for distances higher than 9 Å using the particle-mesh Ewald algorithm [76]. Trajectories were visualized using the VMD software [77] and analyzed using the Curves+ utility [78].

Potential-of-Mean-Force Calculations The free-energy calculations reported herein were carried out utilizing the eABF method [61,62]. To increase the efficiency of the calculations, the free-energy pathway was broken down into up to five consecutive, non-overlapping windows for the different terms of the binding constant. The sampling time required to complete the entire calculation was 971 ns. Instantaneous values of the force were accrued in bins of width equal to $1^{\circ }$, 0.05 Å, and 0.1 Å, for the angular, RMSD, and separation PMFs, respectively. Harmonic angular restraints of netropsin and RMSD restraints of DNA were introduced in each free-energy calculation by means of harmonic potentials with a force constant equal to 0.1 kcal/(mol·degree${}^{2}$) and 100 kcal/(mol·Å${}^{2}$), respectively.

## Figures and Tables

**Table 1:** Contributions to the standard binding free energy for netropsin associated with DNA.

Contribution	PMF (kcal/mol)	Simulation Time (ns)
$\Delta G_{c}^{\mathrm{site}}$	−35.2±1.6	445
$\Delta G_{\Theta }^{\mathrm{site}}$	−0.2±0.0	14
$\Delta G_{\Phi }^{\mathrm{site}}$	−0.1±0.0	10
$\Delta G_{\Psi }^{\mathrm{site}}$	−0.1±0.0	10
$\Delta G_{\theta }^{\mathrm{site}}$	−0.2±0.0	18
$\Delta G_{\varphi }^{\mathrm{site}}$	−0.1±0.0	14
$-\frac{1}{\beta }$ln(S${}^{*}$I${}^{*}$C${}^{\circ }$)	−12.0±0.6	245
$\Delta G_{c}^{\mathrm{bulk}}$	+26.8±1.2	215
$\Delta G_{o}^{\mathrm{bulk}}$	+7.9	-
$\Delta G_{\mathrm{bind}}^{\circ }$	−13.2±2.0	971
$\Delta G_{\mathrm{bind}}^{\circ }$(exp) [58]	−12.7	-
